# Pepper bHLH transcription factor *CabHLH035* contributes to salt tolerance by modulating ion homeostasis and proline biosynthesis

**DOI:** 10.1093/hr/uhac203

**Published:** 2022-09-06

**Authors:** Huafeng Zhang, Jiangbai Guo, Xiaoqing Chen, Yunyun Zhou, Yingping Pei, Lang Chen, Saeed ul Haq, Minghui Lu, Haijun Gong, Rugang Chen

**Affiliations:** College of Horticulture, Northwest A&F University, Yangling 712100, China; College of Horticulture, Northwest A&F University, Yangling 712100, China; College of Horticulture, Northwest A&F University, Yangling 712100, China; College of Horticulture, Northwest A&F University, Yangling 712100, China; College of Horticulture, Northwest A&F University, Yangling 712100, China; College of Horticulture, Northwest A&F University, Yangling 712100, China; College of Horticulture, Northwest A&F University, Yangling 712100, China; Department of Horticulture, The University of Agriculture Peshawar, Peshawar 25130, Pakistan; College of Horticulture, Northwest A&F University, Yangling 712100, China; College of Horticulture, Northwest A&F University, Yangling 712100, China; Shaanxi Engineering Research Center for Vegetables, Yangling 712100, China; College of Horticulture, Northwest A&F University, Yangling 712100, China; Shaanxi Engineering Research Center for Vegetables, Yangling 712100, China

## Abstract

Members of the bHLH family of transcription factors play important roles in multiple aspects of plant biological processes, for instance, abiotic stress responses. Previously, we characterized *CaNAC035*, a gene that positively regulates stress tolerance and identified CabHLH035, a CaNAC035-interacting protein in pepper (*Capsicum annuum* L.). In this study, we describe the role of *CabHLH035* in the response to salt stress. Our results show that the expression of *CabHLH035* increased following salt treatment. Transient expression of *CabHLH035* (*CabHLH035*-To) in pepper enhanced salt tolerance, ectopic expression of *CabHLH035* in Arabidopsis increased the salt stress tolerance, whereas knocking down the expression of *CabHLH035* in pepper plants resulted in decreased salt tolerance. Homologs of the *Salt Overly Sensitive 1* (*SOS1*) and pyrroline-5-carboxylate acid synthetase (*P5CS*) genes showed drastically increased expression in transgenic Arabidopsis plants expressing *CabHLH035* and *CabHLH035*-To plants, but expression decreased in *CabHLH035*-silenced plants. Our results also showed that CabHLH035 can directly bind to the *CaSOS1* and *CaP5CS* gene promoters and positively activate their expression. We found that transgenic Arabidopsis plants, ectopic expression of *CabHLH035* and pepper plants transiently overexpressing *CabHLH035* (*CabHLH035*-To) showed lower Na^+^ and higher proline contents in response to NaCl treatment, while *CabHLH035*-silenced plants had higher Na^+^ and lower proline concentrations. Overall, *CabHLH035* plays important roles in salt tolerance through its effects on the intracellular Na^+^ : K^+^ ratio and proline biosynthesis.

## Introduction

Soil salinity negatively affects crop yields in many parts of the world through threatening crop growth and development. High concentrations of salt (NaCl) can perturb the ion balance in plant cells, causing rising in the concentrations of Na^+^ and Cl^−^ ions. The damage caused by high salt concentrations can impair the ability of plant cells to absorb water and nutrients. High levels of salt lead to severe osmotic stress, which affects many physiological and metabolic processes in plants, causing serious damage and, ultimately, death of the plants. Saline soil has an adverse effect on crop growth by applying ionic toxicity and inhibiting important enzymes [1]. Ionic toxicity is believed to be caused by the Na^+^ transport from roots to shoots [2]. Proline plays pivotal roles in protein synthesis, and has been intensively studied in modulating salt stress. Proline plays pivotal roles and has been intensively studied in modulating salt stress. Proline as a key molecular switches to combat the salt stress, plant salt stress resistance can be effectively improved by higher proline content. [3]. For instance, the *AtMYC2* gene in Arabidopsis is involved in salt tolerance by modulating proline biosynthesis [4]. *FcWRKY40* contributes to salt tolerance by regulating the proline biosynthesis [5]. Basic helix–loop–helix (bHLH) proteins are members of a large super-family of transcription factors (TFs) that are found in eukaryotes from yeast to plants and animals. bHLH TFs play positive roles in regulating plant growth and development, metabolism, and the responses to various stresses. Plant resistance is a complex trait controlled by multiple genes, and it can be affected by other environmental factors. TFs bind directly to specific *cis*-acting elements in gene promoter regions and regulate the expression of downstream genes. TFs can also affect the expression of a series of downstream functional genes by regulating the expression of other transcription factor genes. Therefore, TFs function as key molecular switches in the plant responses to abiotic stresses. bHLHs contain a region of ~60 amino acids that encompasses two functional domains: a basic amino acid domain and a helix–loop–helix (HLH) domain [6]. The basic domain consists of approximately 15 amino acids at the N-terminal end of the 60-amino acid region that allows the TF to bind to G-box (5′-CACGTG-3′) or E-box (5′-CANNTG-3′) elements [7, 8]. The HLH domain is located at the carboy-terminal end of the region and consists of two hydrophobic residues in a helical-ring-helical structure that promote protein–protein interactions [9]. After the MYB TF gene family, bHLHs are the second largest TF gene family in plants. Although the functions of bHLH TFs have been intensively studied in many biological processes, the regulatory model of uncharacterized bHLH TFs remains to be explored.

Many bHLH transcription factor genes have been identified and cloned from different plants; for instance, Arabidopsis [10], tomato [11], rice [12], apple [13], and pepper [14]. It has been reported that bHLH TFs are involved in the growth and development of plants [15] and also modulate iron homeostasis. For example, *SlbHLH068* positively regulated the iron homeostasis in tomato [16]. The tobacco *NtbHLH123* gene is a bHLH-type transcription factor that has been reported to contribute to salt stress [17]. *MfbHLH38*, a bHLH transcription factor gene from the African medicinal shrub *Myrothamnus flabellifolia*, confers tolerance to salt stress tolerance in Arabidopsis [18]. Similarly, *BvbHLH93* is involved in salt stress in Arabidopsis [19]. Arabidopsis *AtUNE12* bHLH TF improves salt stress tolerance by modulating the expression of involved in ion homeostasis [20]. Pepper plants are susceptible to various abiotic (high temperature, cold, high humidity, drought, and salt) and biotic (bacterial wilt, phytophthora, and leaf rolling) stresses, which can result in reduced fruit yield. Thus, it is important to study the resistance mechanisms in pepper by analysing the signaling pathways for genetic improvement of stress tolerance. The functions of individual bHLH TFs in pepper plants are largely unknown. The pepper bHLH family has been characterized, and 122 pepper bHLH protein genes have been identified [14]. In a previously study, we characterized *CaNAC035* in pepper, which positively regulates tolerance to cold, salt, and drought stress tolerance [21]. Using yeast one-hybrid, we identified CabHLH79 bound to the region upstream of the *CaNAC035* promoter [22]; using yeast two-hybrid, we identified CabHLH035, a CaNAC035-interacting protein in pepper. Based on gene-specific expression patterns, the relative expression of *CabHLH035* (*LOC107866727*) increased in response to salt treatment; therefore, we characterized the *CabHLH035* gene and hypothesized that *CabHLH035* plays a crucial role in salt stress tolerance. In the current study, we confirmed the important role of *CabHLH035* in salt tolerance in pepper. These data provide a foundation for studies on the roles of bHLH TFs in pepper and other economically important plant species.

## Results

### Subcellular localization and expression of CabHLH035

**Figure 1:**
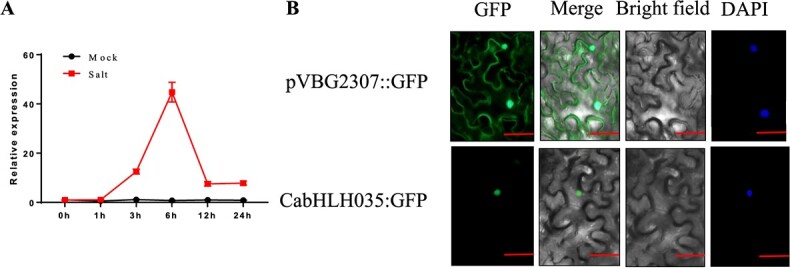
CabHLH035 sub-cellular localization and *CabHLH035* gene expression patterns. **A** Relative transcription of *CabHLH035* in response to 300 mM NaCl treatment at six time points from 0 to 24 hours. **B** CabHLH035 is localized to the nucleus *N*. *benthamiana*. Scale bars = 50 μm. Error bars indicate the standard deviations (SDs) in (**A**).

To investigate the function of *CabHLH035* under condition of salt stress (300 mM NaCl), the expression level of the *CabHLH035* gene was measured using qRT-PCR. Intriguingly, *CabHLH035* expression reached its peak of approximately (43-fold) at 6 h, after which expression rapidly decreased at 12 h (Fig. 1A). These results showed that transcription of *CabHLH035* can rapidly respond to salt treatment. To explore the sub-cellular localization of the CabHLH035 protein, the *Agrobacterium* strains harboring the pVBG2307:*CabHLH035*:GFP and pVBG2307:GFP vectors were infiltrated into leaves of young *Nicotiana benthamiana* plants. The results showed that leaves infiltrated with pVBG2307::GFP had GFP signals in the nucleus and the cell membrane, whereas leaves infiltrated with pVBG2307:CabHLH035:GFP (expressing the CabHLH035:GFP fusion protein) had GFP signals localized to the nuclei only (Fig. 1B). The results of this experiment showed that the CabHLH035 protein localizes to the nucleus in *N*. *benthamiana*.

### Transient expression of *CabHLH035* enhanced pepper salt tolerance

**Figure 2:**
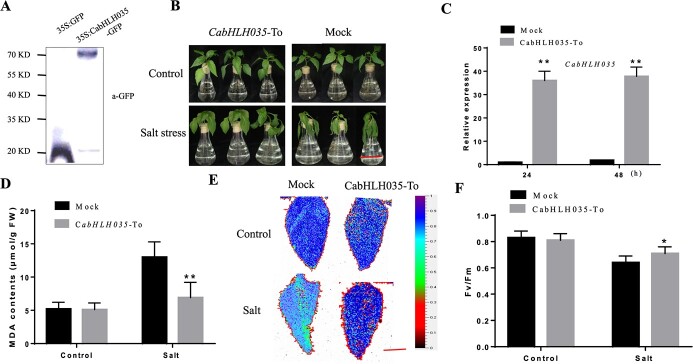
The responses of *CabHLH035*-To and control pepper plants to treatment with 300 mM NaCl. **A***CabHLH035*-GFP transient overexpression in pepper detected by immunoblotting. **B** Phenotypes of *CabHLH035*-To and Mock plants in response to 48 h salt treatment. Scale bars = 2 cm. **C** The expression of *CabHLH035* in *CabHLH035*-To and Mock plants at 24 and 48 h. **D** MDA content in the Mock and *CabHLH035*-To plants after three days of NaCl treatment. **E** Chlorophyll fluorescence images. **F** Fv/Fm values. Scale bars = 1 cm. Error bars denote the standard deviation, and statistically significant differences are denoted by ^*^P ≤ 0.05, ^**^P ≤ 0.01.

To explore the biological function of *CabHLH035* under conditions of salt stress, transient expression of *CabHLH035*-GFP in pepper leaves was performed. Successful expression of *CabHLH035*-To in pepper was authorized by western blot (Fig. 2A). Under normal growth conditions, no significant phenotypic differences were observed between the transient overexpression (*CabHLH035*-To) plants and the 35S::GFP Mock (control) plants. However, after 48 h salt treatment, leaves of the Mock plants showed obvious symptoms of salt stress (wilted and distorted leaves) compared with the *CabHLH035*-To plants (Fig. 2B). In addition, the relative expression level of *CabHLH035* was obviously up-regulated by *CabHLH035*-To compared with the control plant at 24 and 48 h (Fig. 2C). We next investigated the malondialdehyde (MDA) concentration in the *CabHLH035*-To and Mock plants. Without salt conditions, the *CabHLH035*-To and Mock plants showed no differences in MDA content. When the *CabHLH035*-To and Mock pepper plants were exposed to salt treatment for three days, the CabHLH035-To pepper plants showed significantly lower MDA content compared to the Mock plants (Fig. 2D). We also measured the photochemical efficiency of PSII (Fv/Fm) in the *CabHLH035*-To and Mock plants (Fig. 2E). Without salt condition, no conspicuous difference in Fv/Fm was detected in the *CabHLH035*-To and Mock plants. However, after salt stress the Fv/Fm of *CabHLH035*-To pepper plants was obviously higher than in the Mock pepper plants (Fig. 2F). These observations indicate that *CabHLH035*-To increased salt tolerance in pepper.

### Silencing the *CabHLH035* gene decreased pepper salt tolerance

**Figure 3:**
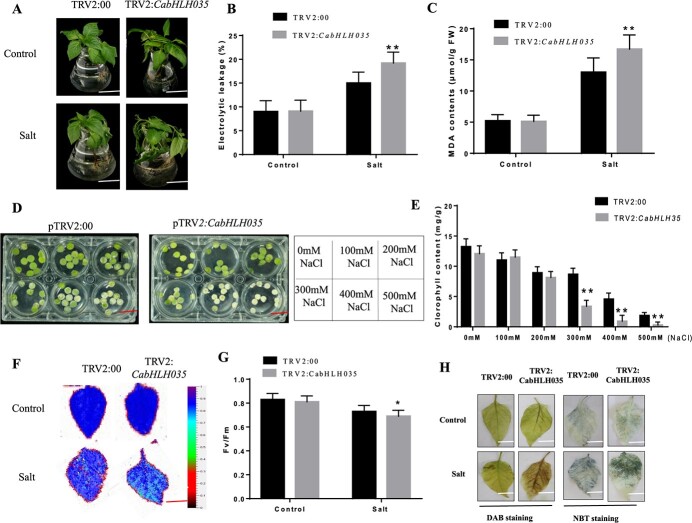
Assessment of *CabHLH035*-silenced pepper plants. **A** Phenotypes of the *CabHLH035*-silenced and empty-vector control plants were subjected to NaCl. Scale bars = 2 cm. **B** REL and **C** MDA content. **D** the phenotypes of leaf disks taken from *CabHLH035*-silenced and the control plants after exposure to different salt contents for three days. **E** Chlorophyll content, **F** Chlorophyll fluorescence images, and **G** Fv/Fm values in leaf disks after three days of treatment with 300 mM NaCl. Scale bars = 1 cm. **H**, DAB and NBT staining for H_2_O_2_ and superoxide anion, respectively. Values are means ± standard deviation (SD). Asterisks (^*^) indicates significant differences (^*^P ≤ 0.05, ^**^P ≤ 0.01).

As shown in Fig. S1 (see online supplementary material), plants infected with the recombinant TRV2:*CaPDS* VIGS vector showed photo-bleaching in the leaves due to knocking down the expression of the phytoene desaturase gene, indicating that virus-induced gene silencing (VIGS) is effective. The silencing efficiency was then measured using qRT-PCR, and was found to be close to 85%. Leaves from the *CabHLH035*-silenced and control plants were immersed in 300 mM NaCl. No differences were observed under normal conditions between the *CabHLH035*-silenced and control plants. After salt stress, the *CabHLH035*-silenced leaves showed obvious wilting compared with leaves of the control pepper plants (Fig. 3A). Leaf disks from the silenced and control plants were floated on six concentrations of NaCl (0, 100, 200, 300, 400, and 500 mM) for three days. No differences were observed between the *CabHLH035*-silenced and the unsilenced control plants at 0 mM NaCl. At the higher NaCl concentrations, the *CabHLH035*-silenced leaf disks showed obvious chlorosis compared to the disks from the control plants (Fig. 3D). Accordingly, the chlorophyll content between the leaf disks from *CabHLH035*-silenced plants and the control plants were similar in the absence of salt. After three days of salt treatment, the chlorophyll contents in the leaf disks from *CabHLH035*-silenced plants were dramatically lower than in leaf disks from the control plants at NaCl concentrations >300 mM, and these differences were statistically significant (Fig. 3E). In addition, there was no evident change in the Fv/Fm of *CabHLH035*-silenced plants under control conditions, while after three days of NaCl treatment, the Fv/Fm of the *CabHLH035*-silenced plants were lower than in the absence of salt (Fig. 3F and G). We also measured the REL and MDA contents. Under normal conditions, the *CabHLH035* VIGS plants and empty-vector control pepper plants showed no obvious differences in their REL and MDA contents. However, when the *CabHLH035-*silenced and the control pepper plants were treated with 300 mM NaCl forthree days, the *CabHLH035*-silenced pepper plants had increasedREL and MDA contents in comparison with the control plants (Fig. 3B and C). In addition, we performed NBT and DAB staining of the leaves to examine the accumulation of reactive oxygen species (ROS) in response to salt stress in the control and silenced pepper plants. Fig. 3H shows that the areas stained by DAB (diaminobenzidine tetrahydrochloride; for H_2_O_2_) andNBT (nitroblue tetrazolium; for superoxide anion) in *CabHLH035*-silenced plants were substantially greater than the control plants. These results show that *CabHLH035*-silenced plants had the highest levels of ROS than the control pepper plants. Overall,our results clearly demonstrate that knocking down *CabHLH035* expression makes pepper plants more sensitive to salt stress.

### Ectopic expression of *CabHLH035* enhanced Arabidopsis salt tolerance

**Figure 4:**
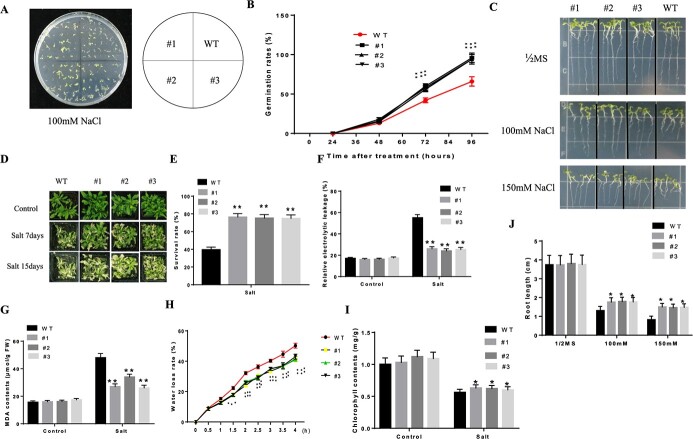
Ectopic expression of *CabHLH035* in Arabidopsis enhances resistance to salt stress. **A**, **B** Germination rates of *CabHLH035* transgenic lines and WT containing 100 mM NaCl. **B**, **C** Greening cotyledon from transgenic and WT containing NaCl. **D** Phenotypes of transgenic Arabidopsis and WT under salt stress. **E** survival rate. **F** REL, **G** MDA contents. **H** Water loss. **I** Chlorophyll contents. **J** Root length. Error bars denote the standard deviation, and statistically significant differences are denoted by ^*^P ≤ 0.05, ^**^P ≤ 0.01.

To further establish the function of *CabHLH035*, *CabHLH035* was ectopically expressed in Arabidopsis. The 35S::*CabHLH035*-GFP vector was generated and introduced into *Agrobacterium* strain GV3101, which was then used to transform Arabidopsis. We selected three *CabHLH035* transgenic lines of Arabidopsis for our experiments, all three lines (#1, #2, and #3) showed higher for the relative expression and survival rate, in comparison with wild-type (WT) plants (Fig. S2, see online supplementary material). We first measured the seed emergence rates of transgenic and WT seeds on 0.5$\times$ Murashige and Skoog (MS) solid medium after 7 days with or without added NaCl. After exposure to 100 mM NaCl, the *CabHLH035* transgenic lines showed obviously higher seed emergence rates than did the WT (Fig. 4A and B). Second, we measured the root lengths of seedlings on MS medium containing 0, 100, and 150 mM NaCl (Fig. 4C and J), and no obvious changes were observed between the WT and transgenic plants without salt stress. After treatment with 100 and 150 mM NaCl, the roots of the *CabHLH035* transgenic lines were substantially longer than in WT seedlings. Third, we checked the response of mature plants to salt stress. As shown in Fig. 4D, after 300 mM salt treatment for seven days, the leaves of the transgenic line seedlings showed little yellowing, while WT leaves wilted and the plants ultimately died. Similarly, 15 days after NaCl treatment (300 mM), all the WT plants showed severe wilting compared to the *CabHLH035* transgenic plants, as indicated by the higher survival rate of the *CabHLH035* transgenic plants (Fig. 4E). Meanwhile, the chlorophyll content of *CabHLH035* transgenic Arabidopsis plants was prominently higher than that of WT plants (Fig. 4I). Following salt treatment, the *CabHLH035* transgenic lines exhibited markedly increased salt tolerance, accompanied by lower REL and MDA contents (Fig. 4F and G). To further determine whether the enhanced response to salt stress in *CabHLH035* transgenic Arabidopsis plants is due to decreased transpiration, we measured the rate of water loss in 3-week-old transgenic and WT plants. As shown in Fig. 4H, in comparison with WT, the *CabHLH035* transgenic lines had substantially lower water loss rates. Overall, these results show that the *CabHLH035* gene positively regulates salt stress tolerance.

### The transcripts of genes related to intracellular Na^+^ : K^+^ ratio and proline synthesis

**Figure 5:**
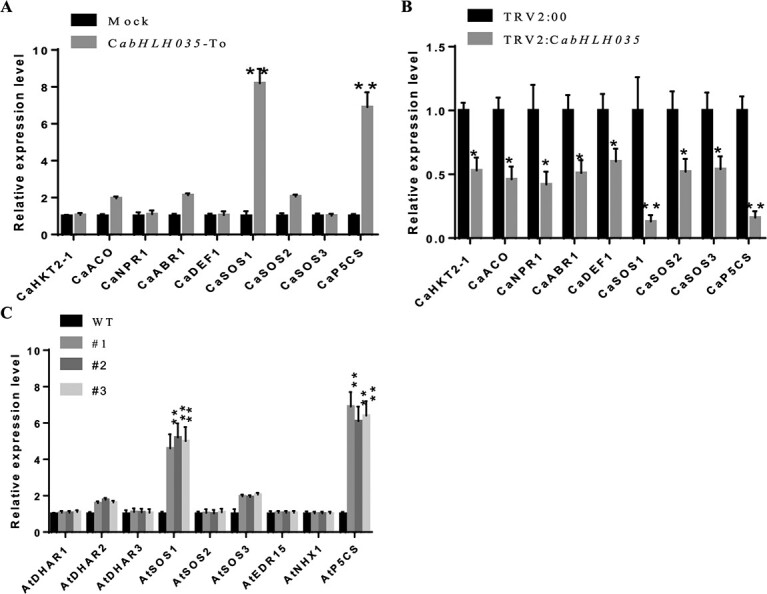
Relative transcription of genes related to intracellular Na^+^ : K^+^ ratio and proline synthesis. **A** Expression of ion homeostasis and proline synthesis genes in *CabHLH035*-To and control pepper plants. **B** The expression of ion homeostasis and proline synthesis genes in *CabHLH035*-silenced and control plants. **C** The expression of ion homeostasis and proline synthesis genes in three transgenic *CabHLH035*-overexpressing Arabidopsis lines and WT plants. Error bars show ± SD (*n* = 3). Asterisks indicate significant differences (^*^P ≤ 0.05, ^**^P ≤ 0.01).

To elucidate the function of *CabHLH035* in response to salt tolerance, we studied the expression of salt stress-related genes, including *SALT OVERLY SENSITIVE 1–3* (*SOS1, SOS2,* and *SOS3*) and *HIGH-AFFINITY K^+^ TRANSPORTER 2–1* (*HKT2–1*), the defense-related genes *AMINOCYCLOPROPANE-1-CARBOXYLATE OXIDASE* (*ACO*), *NONEXPRESSER of PR GENES 1* (*NPR1*), *CaABR1*, and *DEFENSIN 1* (*DEF1*)*,* and the proline synthesis-related gene pyrroline-5-carboxylate acid synthetase (*P5CS*) in *CabHLH035* transgenic Arabidopsis plants, *CabHLH035*-To transient expression plants, and *CabHLH035*-silenced plants. These genes were related to ion equilibrium or proline concentration, and have been identified as being positive regulators of salt tolerance [23–25]. We found that among the tested genes, the expression patterns of *SOS2*, *SOS3, HKT2–1*, *ACO*, *NPR1*, *ABR1*, and *DEF1* did not change in the *CabHLH035* transgenic Arabidopsis, *CabHLH035*-To, and *CabHLH035*-silenced plants. However, the relative expression of *SOS1* and *P5CS* was prominently lower in the *CabHLH035* VIGS plants than in the control pepper plants. In contrast, the *SOS1* and *P5CS* mRNA levels of *CabHLH035*-overexpressing and *CabHLH035*-To plants were dramatically higher than the control plants (Fig. 5).

### The accumulation of Na^+^, K^+^ and proline concentration

**Figure 6:**
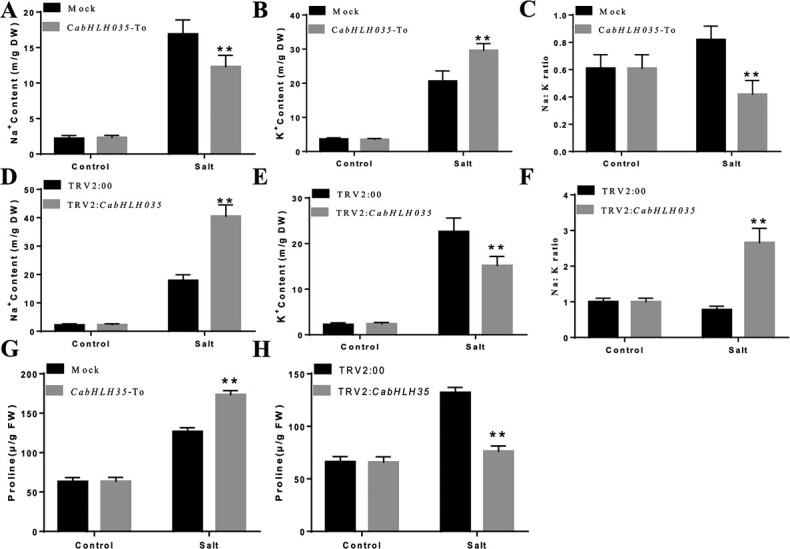
Analysis of Na^+^, K^+^, and proline contents in *CabHLH035*-silenced and *CabHLH035*-To pepper plants after NaCl treatment. Contents of Na^+^ (**A**, **D**), K^+^ (**B**, **E**), Na^+^ : K^+^ ratios (**C**, **F**) and endogenous proline concentrations (**G**, **H**) in the leaves of *CabHLH035*-silenced, *CabHLH035*-To pepper, and control pepper plants. Error bars denote the standard deviation, and statistically significant differences are denoted by (^*^P ≤ 0.05, ^**^P ≤ 0.01).

Previous studies have shown that the SOS pathway plays a central role in maintaining cellular ion homeostasis; the SOS pathway is responsible for Na^+^ homeostasis in plants [26]. Proline has been shown to modulate abiotic stresses, especially salt stress [27]. We determined the Na^+^ and K^+^ concentrations in *CabHLH035*-To and *CabHLH035*-silenced plants, but no clear differences in the Na^+^ and K^+^ concentrations were found between them and the control pepper plants. However, salt stress caused severe leaf wilting and resulted in higher Na^+^ concentrations and lower K^+^ concentrations in *CabHLH035*-silenced plants compared to the controls (Fig. 6D and E). In addition, the Na^+^ : K^+^ ratios were markedly increased in the *CabHLH035*-silenced plants (Fig. 6F). In contrast, the *CabHLH035*-To plants had lower Na^+^ and higher K^+^ contents than the control pepper plants (Fig. 6A and B). In addition, the Na^+^ : K^+^ ratios were significantly down-regulated in the *CabHLH035*-To plants (Fig. 6C). Next, we measured the proline contents, and found that the proline content of *CabHLH035*-silenced plants showed decreased proline content compared with the control plants (Fig. 6H), and the *CabHLH035*-To plants had higher proline contents than did the control plant under salt stress conditions (Fig. 6G). These results show that the tolerance of *CabHLH035*-To plants and the susceptibility of *CabHLH035*-silenced plants to salt stress were associated with the intracellular Na^+^ : K^+^ ratio and proline concentration.

### *CaSOS1* is a direct target gene of CabHLH035

**Figure 7:**
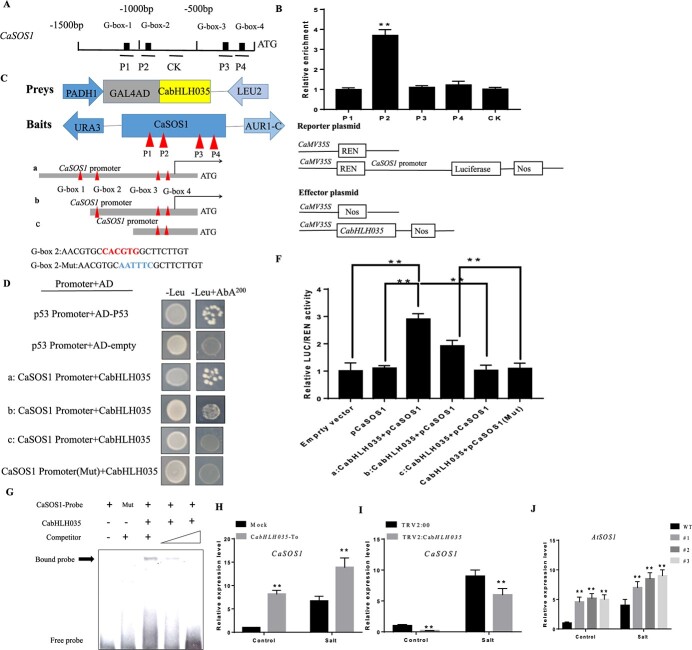
*CaSOS1* is a target gene of CabHLH035 under salt stress conditions. **A** Diagrams showing the *CaSOS1* promoter and the four G-box elements. **B** ChIP assays of CabHLH035 binding to *CaSOS1* promoter. **C** the bait and prey DNA constructs used in the Y1H assays. **D** Results of the Y1H experiment. **E** Schematic diagrams of the effector and reporter constructs used in the luciferase experiment. **F** LUC/REN ratios. **G** EMSA analysis of CabHLH035 binding to the *CaSOS1* promoter. **H**, **I***CaSOS1* expression is up-regulated by transient overexpression but is down-regulated when *CabHLH035* is silenced in pepper leaves exposed to salt stress. **J***AtSOS1* was up-regulated in transgenic Arabidopsis lines overexpressing *CabHLH035*. REN is the luciferase gene from *Renilla.* P1–P4 is the fragment amplified by primer pairs and CK is the G-box-free fragments. Error bars show the standard deviation (SD). Asterisks indicate statistical significance (^*^P ≤ 0.05, ^**^P ≤ 0.01).

We observed that the transcription of the endogenous *SOS1* genes were dramatically changed in *CabHLH035* transgenic Arabidopsis plants and *CabHLH035*-To and *CabHLH035*-silenced pepper plants. Previous studies have shown that G-box or E-box motifs bound by bHLH proteins. We found that the sequences of the promoter regions upstream of the pepper CaSOS1 gene contain four *cis*-acting G-box elements (Fig. 7A). The ChIP-qPCR results showed that the P2 fragment of the *CaSOS1* promoter had a higher relative enrichment, which suggested that the CabHLH035 protein is able to bind the P2 fragment of the CaSOS1 promoter region (Fig. 7B). To show that the CabHLH035 protein can directly bind to the CaSOS1 P2 promoter fragment, yeast one-hybrid (Y1H) was carried out. The Y1H results showed that yeast cells co-expressing the prey and bait plasmids grew normally on the selective media, suggesting that CaSOS1 interacts with CabHLH035 (Fig. 7C and D). We then performed Dual LUC (luciferase) assays to test the interaction between CaSOS1 and CabHLH035. The LUC/REN ratio of the a, b:CabHLH035 + paCaSOS1 sample was higher than other samples, which strongly suggests that the CabHLH035 TF protein binds to the CaSOS1 gene promoter region (Fig. 7E and F). An electrophoretic mobility shift assay (EMSA) also indicated that *CaSOS1* is a direct target of CabHLH035*,* as revealed by the *CabHLH035*-probe DNA complex on the polyacrylamide gel (Fig. 7G). Based on the LUC/REN, ChIP-qPCR, Y1H, and EMSA results, when the G-box 2 was mutated, CaSOS1 not interacts with CabHLH035. These results CabHLH035 bind to the *CaSOS1* G-box 2 promoter fragment. As was evident when the two genes were co-expressed, the *CaSOS1* mRNA level decreased in *CabHLH035*-silenced pepper plants exposed to salt stress and was obviously higher in plants in which *CabHLH035* was transiently overexpressed (Fig. 7H and I). Similarly, in comparison with WT, the *AtSOS1* mRNA level was higher in three transgenic *CabHLH035*-OE Arabidopsis lines (Fig. 7J). These results indicate that *CaSOS1* is a direct target that is positively regulated by CabHLH035 under condition of salt stress.

### CabHLH035 interacts with the promoter of *CaP5CS*

**Figure 8:**
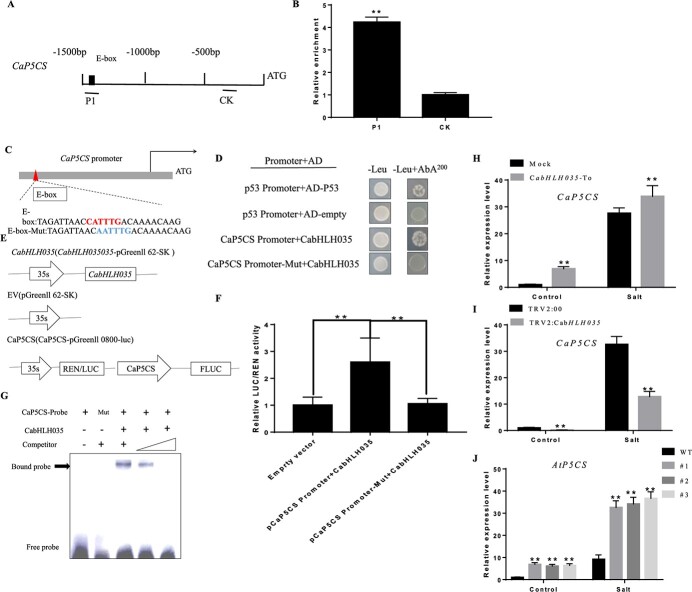
*CaP5CS* is a target gene of CabHLH035. **A**, **C** Schematic diagrams of the *CaP5CS* gene promoter region showing the position and sequence of the E-box element. **B** A ChIP analysis of CabHLH035 to the *CaP5CS* promoter. **D** Yeast-one hybrid (Y1H) analysis of the physical interaction between CabHLH035 and the *CaP5CS* gene promoter. **E** Diagrams showing the maps of the effector and reporter plasmid constructs. **F** LUC/REN ratios. **G** EMSA showing that CabHLH035 binds to the *CaP5CS* promoter region. **H**–**J** Relative expression levels of *CaP5CS* in pepper leaves transiently overexpressing *CabHLH035*, in pepper leaves in which *CabHLH035* was silenced using VIGS, and expression of *AtP5CS* in three transgenic Arabidopsis lines overexpressing *CabHLH035*. P1 is the fragment amplified by primer pairs and CK is the E-box-free fragments. Error bars show the standard deviation (SD). Asterisks indicate statistical significance (^*^P ≤ 0.05, ^**^P ≤ 0.01).

We examined the expression level of the proline synthesis gene *P5CS* in pepper plants exposed to NaCl. The P5*CS* changed in *CabHLH035*-expressing transgenic Arabidopsis plants and *CabHLH035*-To and *CabHLH035*-silenced pepper plants. We hypothesized that CabHLH035 directly regulates *CaP5CS* gene transcription. Our analysis of the DNA sequence upstream of CaP5C revealed the presence of an E-box element (Fig. 8A). A higher relative enrichment of the E-box DNA fragment from the CaP5CS promoter was found, followed the ChIP-qPCR, suggesting that CabHLH035 binds to the E-box element present in the CaP5CS gene promoter (Fig. 8B). To further determine whether CabHLH035 can directly bind to the CaP5CS promoter region, we performed a Y1H assay. The results showed that yeast cells in which the prey and bait plasmids were co-expressed grew normally on the selective media. Nevertheless, when the E-box was mutated, the yeast did not grow on the selective media (Fig. 8C and D). To further establish this relationship, we measured the LUC/REN ratios, and tested whether CaP5CS is a direct target gene of CabHLH035. The results strongly suggest that the CabHLH035 TF binds to the CaP5CS gene promoter region (Fig. 8E and F). EMSA analysis revealed that *CaP5CS* is a direct target of CabHLH035*,* as revealed by the protein DNA complex between CabHLH035 and the *CaP5CS* probe observed on a polyacrylamide gel (Fig. 8G). Based on the LUC/REN, ChIP-qPCR, Y1H, and EMSA results, when the E-box was mutated, CaP5CS does not interact with CabHLH035. These results show CabHLH035 binds to the *CaP5CS* E-box promoter fragment. To explore the relationship between *CabHLH035* and *CaP5CS*, we measured the mRNA level of *P5CS* in *CabHLH035*-overexpressing transgenic Arabidopsis plants and *CabHLH035*-To and *CabHLH035*-silenced pepper plants. We found that the *P5CS* mRNA level was higher in *CabHLH035* transgenic Arabidopsis and *CabHLH035*-To pepper plants compared to the control plants (Fig. 8H and J). In contrast, the *CabHLH035* VIGS plants decreased expression of *CaP5CS* (Fig. 8I). These results indicate that CabHLH035 interacts with the promoter of CaP5CS.

### Effect of *CaNAC035* silencing on the binding of CabHLH035 to the G-or E-box containing promoters of *CaSOS1* and *CaP5CS*

**Figure 9:**
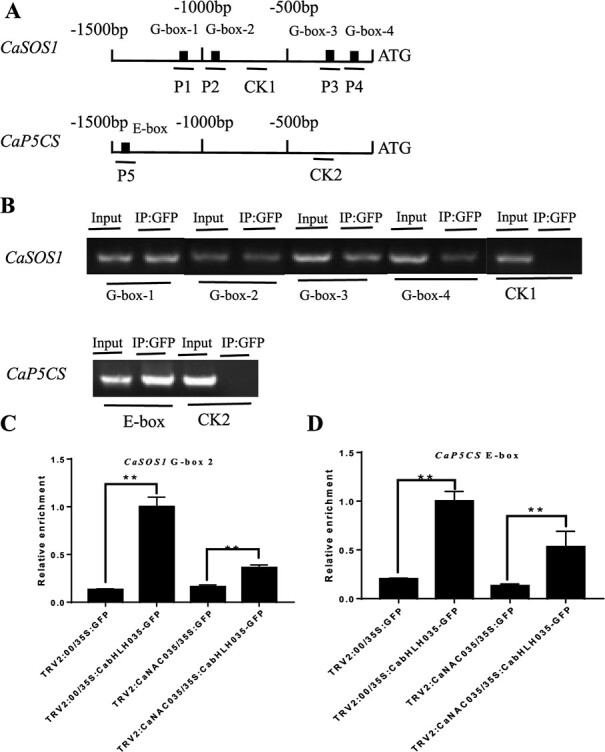
Effect of silencing the CaNAC035 gene on CabHLH035 binding to the *CaSOS1* and *CaP5CS* gene promoter regions. **A** Schematic diagrams of the *CaSOS1* and *CaP5CS* gene promoters showing the locations of the G- and E-box elements. P1–P5 are the primers used to amplify the specific DNA sequences. **B***ChIP-PCR* analysis of the binding of *CabHLH035* to the G-box- or E-box-containing promoters of *CaSOS1* and *CaP5CS*. **C**, **D***ChIP-qPCR* analysis of *CaNAC035* silencing on the binding of *CabHLH035* to the G-boxes in the *CaSOS1* promoter and the E-box in the *CaP5CS* promoter. Error bars show the standard deviation (SD). Asterisks indicate statistical significance (^*^P ≤ 0.05, ^**^P ≤ 0.01).

In this study we found that CabHLH035 can bind to the promoter motifs of the *CaSOS1* and *CaP5CS* genes. To determine whether *CaSOS1* and *CaP5CS* are directly targeted by CabHLH035, and if so, if the targeting is regulated by CaNAC035, the chromatin from 35S:*CabHLH035*:GFP in *CaNAC035*-silenced and TRV2:00 (control) plants were assayed by ChIP-PCR and qPCR. The DNA of ChIP was used as the template in PCR amplification containing the G-and E-box sequences (Fig. 9A). The ChIP-PCR results indicated that CabHLH035 could bind to the *CaSOS1* and *CaP5CS* promoter regions (Fig. 9B). *CaNAC035* silencing reduced CabHLH035 binding to the *CaSOS1* and *CaP5CS* promoters (Fig. 9C and D). The results show that *CaNAC035* is requisitioned for the targeting of CabHLH035 binding to G-box or E-box elements in the *CaSOS1* and *CaP5CS* promoters under salt stress.

### *CabHLH035*-mediated salt tolerance via proline accumulation pathway

**Figure 10:**
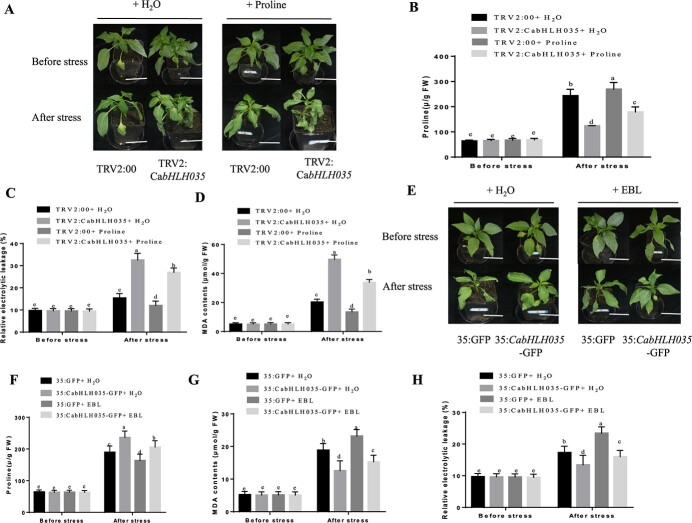
Phenotypes of plants sprayed with exogenous proline and treated with 24-epibrassinolide (EBL). **A** Phenotypes of water- or proline-pretreated *CabHLH035*-silenced and control pepper plants. Scale bars = 2 cm. **B**, **F** proline concentrations in *CabHLH035-*silenced VIGS plants treated with water or proline (**B**) and plants transiently overexpressing *CabHLH035* treated with water or EBL (**F**). **C**, **H** REL in *CabHLH035-*silenced VIGS plants treated with water or proline (**C**) and plants transiently overexpressing *CabHLH035* treated with water or EBL (**H**). **D**, **G** MDA contents in *CabHLH035-*silenced VIGS plants treated with water or proline (**D**) and plants transiently overexpressing *CabHLH035* treated with water or EBL (**G**). **E** Phenotypes of water- or EBL-treated *CabHLH035*-To and control pepper plants. Scale bars = 2 cm. Error bars show the standard deviation, the small letters show significant differences at *P* ≤ 0.05.

The relative expression of *P5CS* changed in *CabHLH035*-To (transiently overexpressing) and *CabHLH035*-silenced pepper plants (Fig. 5), indicating that CabHLH035-mediated salt tolerance via proline accumulation. We therefore measured the endogenous proline contents of *CabHLH035*-To plants treated with 0.05 μM 24-epi-brassinolide (EBL), a proline biosynthesis inhibitor that has been shown to reduce proline content [28] and *CabHLH035*-silenced plants that were sprayed with a 5 mM proline solution, and observed their responses to salt stress. No dramatic differences in proline, REL, and MDA contents were found in the *CabHLH035*-silenced and control plants under normal conditions. Under salt stress, the proline, REL, and MDA contents were increased; however, plants treated with exogenous proline had significantly lower REL and MDA contents and higher proline contents (Fig. 10B–D). These results suggest that exogenous application of proline restored salt tolerance to the *CabHLH035*-silenced plants, further illustrating the importance of proline in *CabHLH035*-mediated salt tolerance in pepper. There were no differences in proline, REL, and MDA contents in the *CabHLH035*-To and control plants before exposure to salt stress. However, the proline, REL, and MDA contents were markedly increased in response to salt stress, and plants treated with EBL had higher REL and MDA levels and lower proline contents than the water-treated controls (Fig. 10E–H). These results show that a reduction in the proline content in *CabHLH035*-To pepper plants significantly decreased their salt tolerance.

## Discussion

Because of the sessile life habit of plants and variable environmental conditions, plants must adapt to a range of environmental challenges. Saline soils are a major abiotic stress that impedes plant growth, crop yields, and farmers’ incomes [29]. Members of the bHLH family of transcription factors (TFs) are involved in many biological processes and the response to abiotic stress in plants. A recent study identified 122 bHLH TF family members in pepper (*Capiscum annuum*) [14], but only a few that regulate plant responses to various stresses have been characterized. In a previous study we characterized the pepper gene *CaNAC035*, which positively regulates stress tolerance. We showed that CabHLH79 binds to the *CaNAC035* promoter, and CabHLH79 could enhance cold tolerance [21]. We also identified CabHLH035, a CaNAC035-interacting protein in pepper [22]. Based on mRNA expression levels, we found that transcription of *CabHLH035* is induced by NaCl treatment (Fig. 1). In our previous study, *CaNAC035* was a positive regulator for cold, mannitol, and salt stress. Many studies have reported that NAC TFs play important roles in plants in processes that include leaf senescence, secondary metabolism, and responses to various unfavorable environmental conditions [30–33]. In this study, we characterized the role of *CabHLH035* TF gene from pepper leaves, and show that it responds to salt stress (Fig. 1). The results of our study provide new insight into *CabHLH035* in response to salt stress.

Reactive oxygen species (ROS) is associated with many abiotic stresses in plants [34]. Production of high levels of ROS leads to oxidative damage in cells, especially under abiotic stress conditions [35]. We found that *CabHLH035*-silenced plants had substantially higher MDA levels than in control plants (Fig. 3). A better explanation might be that *CabHLH035* protects plants from oxidative damage by scavenging ROS. Overall, our findings suggest that *CabHLH035* contributes to salt tolerance.

Chlorophyll content is reduced when plants are subjected to adverse conditions [36]. In our study, the chlorophyll contents of *CabHLH035*-silenced pepper plants were lower compared to non-silenced plants at NaCl concentrations >200 mM (Fig. 3). In plants, MDA content is an indicator of ROS-mediated damage to cell membranes. Ion leakage is another significant physiological indicator of membrane damage [37]. Following salt treatment, in comparison with WT, the transgenic *CabHLH035* Arabidopsis lines showed significantly lower REL and MDA levels (Fig. 4). These data reveal that *CabHLH035* plays a role in mediating salt stress tolerance. Compared with *CabHLH035*-silenced plants, *CabHLH035*-To plants affected less gene expression in Fig. 5; perhaps ectopic expression of *CabHLH035* in Arabidopsis was not high enough. The amino acid proline has been reported to modulate abiotic stresses in plants, especially salt stress. Thus, the proline accumulation is considered to be an important mechanism in plant salt stress tolerance [38]. The proline content of *CabHLH035*-silenced plants was dramatically reduced after salt treatment, however, and the *CabHLH035-*To plants had higher proline contents than the control plants (Fig. 6). It is worth noting that the proline content showed a positive correlation with salt tolerance. Thus, we speculated that the proline concentration had an effect on the response of the *CabHLH035* gene to salt stress. *CaP5CS* is a proline biosynthesis gene, and we found that that after salt treatment, the *CabHLH035*-silenced plants showed lower *CaP5CS* gene expression levels than did the control plants (Fig. 8). However, the *CabHLH035* transgenic Arabidopsis lines showed higher *AtP5CS* gene expression levels compared to WT (Fig. 8). We should note that the expression pattern of *P5CS* was consistent with the proline contents in the plants, suggesting that the free proline content may depend on the relative level of *P5CS* gene expression. To further explore how *CabHLH035* positively regulates salt stress, expression of the proline synthesis gene *P5CS* was measured, and we found that *P5CS* mRNA levels increased in *CabHLH035* transgenic and *CabHLH035*-To lines but decreased in *CabHLH035*-silenced plants (Fig. 5). To investigate whether CabHLH035 binds to the *CaP5CS* promoter region to directly regulate salt resistance, we performed Y1H, Dual LUC, and EMSA assays. The results showed that CabHLH035 binds to the *CaP5CS* promoter, resulting in increased salt tolerance (Fig. 8). We therefore hypothesize that the bHLH-P5CS pathway mediates and the *CabHLH035* gene contributes to salt.

Salt stress severely limits plant growth, development, and yield, and it has two main adverse effects on plants: ion toxicity and osmotic stress [39]. Maintaining an optimal Na^+^ : K^+^ ratio is an important way to alleviate ion toxicity and is necessary to maintain plant natural habitats [40], which suggests that ion homeostasis is the main determinant of salt tolerance. In this study, *SOS* genes, especially *SOS1*, showed higher expression levels in the *CabHLH035* transgenic and *CabHLH035*-To plants, but lower expression in the *CabHLH035*-silenced plants (Fig. 5). We found that the *CabHLH035*-silenced pepper had higher Na^+^ and lower K^+^ contents than did the control pepper plants under salt stress, which led to increased Na^+^ : K^+^ ratios. The *CabHLH035*-To lines had lower Na^+^ and higher K^+^ contents, along with lower Na^+^ : K^+^ ratios, than the control pepper plants (Fig. 6). These results show that salt tolerance in the *CabHLH035* transgenic and *CabHLH035*-To plants and the increased salt susceptibility of *CabHLH035*-silenced plants is primarily due to the intracellular ion balance, therefore lower Na^+^ : K^+^ ratios confer increased salt tolerance. Thus, it is conceivable that changes in the *SOS* genes are key to the accumulation of intracellular Na^+^ and the growth performance of plants under salt stress. We observed that there is a positive correlation between the *CabHLH035* response to salt tolerance and the expression levels of *CaSOS1*. Based on the Y1H, LUC/REN ratios, and EMSA test assays, we conclude that *CaSOS1* expression is regulated by CabHLH035, which indicates that *CaSOS1* is a target gene of CabHLH035 (Fig. 7). Therefore, *CabHLH035* plays an important role in maintaining the intracellular Na^+^ balance, which indicates that *CaSOS1* is critical for growth in plants exposed to salt stress. In summary, we have shown that CabHLH035 regulates *CaSOS1* expression and thus acts as a major regulator of salt tolerance.

## Conclusion

**Figure 11:**
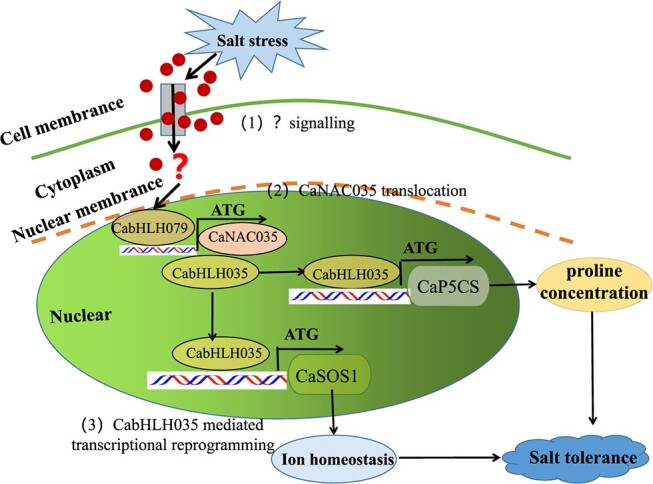
A proposed model showing how *CabHLH035* functions in the plant response to salt stress. Upon salt stress, *CabHLH079* is activated, CaNAC035 is a direct target gene of CabHLH079, and CaNAC035 interact with CabHLH035, to regulate *CaSOS1* and *CaP5CS* by binding to G-Box or E-Box motifs. Up-regulation of the CabHLH035-CaSOS1/CaP5CS modulates ion homeostasis and proline biosynthesis to achieve salt tolerance.

We identified *CabHLH035* was induced by salt treatment. Silencing the *CabHLH035* gene through VIGS decreased the pepper salt tolerance. However, transient expression of *CabHLH035* enhanced pepper salt tolerance and ectopic expression of *CabHLH035* enhanced Arabidopsis salt tolerance. *CaSOS1* and *CaP5CS* are the direct target genes of CabHLH035. Herein, we proposed the model for *CabHLH035* in response to salt stress (Fig. 11). Our study provides a basis for further study to unravel the molecular mechanism of *CabHLH035* in response to salt stress and the stress-related candidate genes to utilize it in molecular breeding to enhance the salt tolerance in pepper and other important crops.

## Materials and methods

### Plant material and growth conditions

In this study, pepper cultivar P70 and *Arabidopsis thaliana* (ecotype Columbia-0) seedlings were used in this study. The test plants were grown at 25°C in daylight for 16 h and 18°C in dark for 8 h with 80% humidity. In order to examine the expression of *CabHLH035*, one-month-old uniform size pepper seedlings were transferred to 300 mM NaCl solution for 24 h. The tissues were collected at 0, 1, 3, 6, 12, and 24 h of 300 mM NaCl treatment. All materials were independently harvested at the specified time.

### RNA extraction and qRT-PCR analysis

Total RNA was extracted as described by Prime Script Kit (Takara, Dalian, China). First-strand cDNA was obtained following the manufacturer protocol of Prime Script Kit (Takara). qRT-PCR was measured using the iCycleriQ™ (Bio-Rad, Hercules, CA, USA) machine. The gene expressions were quantified by the 2^-ΔΔCt^ method, all the primers used for this study are listed in Table S1 (see online supplementary material).

### Subcellular localization and transient expression of CabHLH035

The coding sequence of *CabHLH035* was amplified using gene-specific primers, then introduced into the pVBG2307::GFP vector to construct the pVBG2307:*CabHLH035*:GFP plasmid; the pVBG2307::GFP vector without *CabHLH035* was used as control. The designed construct was infiltrated into *Nicotiana benthamian*a leaves and were grown at 22/18°C (day/night) for two days and the *CabHLH035* was visualized through a fluorescent microscope (Olympus, Tokyo, Japan). The transient expression in pepper was carried out as described by Cai *et al.* (2021) [ 41].

### Virus-induced gene silencing (VIGS)

Through VIGS the *CabHLH035* was knocked-down in pepper as previously described by Lim *et al.* (2015) [42]. A 300-bp fragment of *CabHLH035* was introduced into the vector pTRV2, which is driven by a CaMV 35S promoter. The pTRV1, pTRV2:00, and the fusion plasmids of pTRV2:*CabHLH035* were separately transformed into *Agrobacterium* strain GV3101 and co-infiltrated into the leaves of pepper seedlings. One month later, fully expanded uniformly sized leaves were harvested from the infected plants for silencing efficiency by qRT-PCR and other biochemical analyses.

### Generation of transgenic Arabidopsis

To generate *CabHLH035*-overexpressing Arabidopsis, the coding sequence (CDS) of *CabHLH035* was introduced into the pVBG2307-GFP vector. The fusion constructs of 35S:CabHLH035-GFP were transformed into GV3101. The floral dip method was used for genetic transformation [43]. For the selection of *CabHLH035* transgenic Arabidopsis lines, seeds were selected on MS medium containing 50 mg/L kanamycin. The T3 plants were selected for further study.

### Salt tolerance assay

To analyse the salt tolerance in pepper plants, leaf samples of the transient expression *CabHLH035* (*CabHLH035*-To), 35S::GFP (Mock), *CabHLH035*-silenced pepper plants were immersed in 300 mM NaCl concentrations for three days. To further identify the role of *CabHLH035* in salt stress tolerance of the transgenic Arabidopsis, the three-week-old WT and T3 transgenic lines were treated with 300 mM NaCl. The samples for gene expression analyses were taken on the seventh day and the phenotypic changes were observed and photographed at the seventh and fifteenth days. Samples for the stress-related genes expression analysis and physiological indices were determined after treatment with salt. The control plants were grown in distilled water. Each line contained 30 plants for every test.

### Biochemical analyses and histochemical staining

The relative electrolyte leakage (REL) was assessed according to the method of Cao *et al.* (2007) [44]. The MDA content was measured using the thiobarbituric acid (TBA) method as described by Heath and Packer (1968) [45]. Briefly 0.5 g leaves were grounded in 1 mL 10% trichloroacetic acid (TCA) then diluted with TCA to 10 mL, 12 000 g, for 10 min at 4°C, the supernatant was mixed with 0.6% 2-thiobarbituric acid (TBA) and boiled for 15 min. Absorbance was measured at 600, 532, and 450 nm.

Chlorophyll fluorescence was determined using an IMAGING-PAM chlorophyll fluorimeter, and Fv/Fm ratios were estimated using Imaging software. The total chlorophyll content was determined using the methods of Arkus *et al.* (2005) [46]. Histochemical staining of O_2_^−^ and H_2_O_2_ was executed with NBT and DAB, respectively, following the methods of Huang *et al.* (2013) [47]. For water loss rate, the leaves from 4 weeks of *CabHLH035* transgenic and WT plants were collected and respectively weighed. At least 30 leaves of each *CabHLH035* transgenic and WT plants were weighed every 30 minutes, the water loss rate was assessed following the method of Wei *et al.* (2018) [48]. Proline contents were quantified according to the method of Zhuo *et al.* (2018) [49]. The leaves of ectopic expression of *CabHLH035* in Arabidopsis plants, *CabHLH035*-To transient expression plants, *CabHLH035*-silenced plants, and control plants were collected, then dried at 80°C for 48 h, and the Na^+^ and K^+^ contents were examined as described by the method of Li (2015) [50].

### Yeast-one-hybrid assay

Promoter sequences (1500 bp upstream of ATG) were acquired from the pepper genome platform (PGP). The CDS of CabHLH035 was inserted into the pGADT7, and promoter sequence fragments of *CaP5CS* and *CaSOS1* were inserted into the pAbAi vector. Recombinant vectors were co-transformed into Y1H and the Y1H experiment was performed following the Clontech method. The bait yeast strain was coated on the SD/−Leu/ medium, and incubated at 30°C for 5–7 days.

### Dual luciferase and electrophoretic mobility shift (EMSA) assay

Dual luciferase was calculated by determining the ratio of luciferase (LUC) and Renilla (REN) using the Dual-Luciferase® machine (Promega, WI, USA). The coding sequence of CabHLH035 was inserted into the pMAL-c5x vector and then transformed into *Escherichia coli* BL21. The mixture was incubated with binding buffer at 24°C for 30 minutes. Protein-probe was separated by the PAGE method. EMSA was performed following the methods of Xie *et al.* (2018) [51].

### Chromatin immunoprecipitation (ChIP) assay

ChIP experiment was carried out according to the method of Khan *et al.* (2018) [52]. The 35S:CabHLH035-GFP was transformed into GV3101 then infected the pepper leaves. The infected pepper leaves were collected at 48 hpi and cross-linked with 1% formaldehyde and the chromatin was divided into 500 bp fragments. DNA-protein complexes were immunoprecipitated with GFP antibodies, and DNA fragments were decross-linked and purified, the template was used for ChIP-qPCR using specific primer of the G-box-or E-box fragments in CaSOS1 and CaP5CS promoter (Table S1, see online supplementary material).

### **Statistical analysi**s

The statistical analyses were performed using one-way analysis of variance (ANOVA) tests with least significant differences at *P* ≤ 0.05 (^*^) and *P* ≤ 0.01 (^**^) by the statistical package SPSS 17.0 (IBM, Chicago, IL, USA).

## Acknowledgments

This research was supported by the National Natural Science Foundation of China (32172582, 31672146), Agricultural Key Science and Technology Program of Shaanxi Province (2021NY-086), Scientific & Technological Innovative Research Team of Shaanxi Province (2021TD-34), and the Natural Science Foundation of Shaanxi Province (2018JM3023).

## Author contributions

H.Z., R.C., and H.G. conceived the experiments; H.Z., Y.P., M.L., and L.C. performed the experiments; U.S., J.G., Y.Z., and X.C. analysed the data; H.Z. wrote the paper.

## Data availability

The data that support the results are included in this article and its supplementary materials.

## Conflict of interest

The authors declare no conflict of interest.

## Supplementary data

Supplementary data is available at *Horticulture Research* online.

## Supplementary Material

Web_Material_uhac203
