# Supplementary material for: Pepper bHLH transcription factor CabHLH035 contributes to salt tolerance by modulating ion homeostasis and proline biosynthesis
Source: Hortic Res. 2022 Sep 6;9:uhac203. doi: 10.1093/hr/uhac203 (PMC9634760; doi:10.1093/hr/uhac203)
Supplement: Web_Material_uhac203 [file web_material_uhac203.docx]

Supplementary materials:

Figure S1


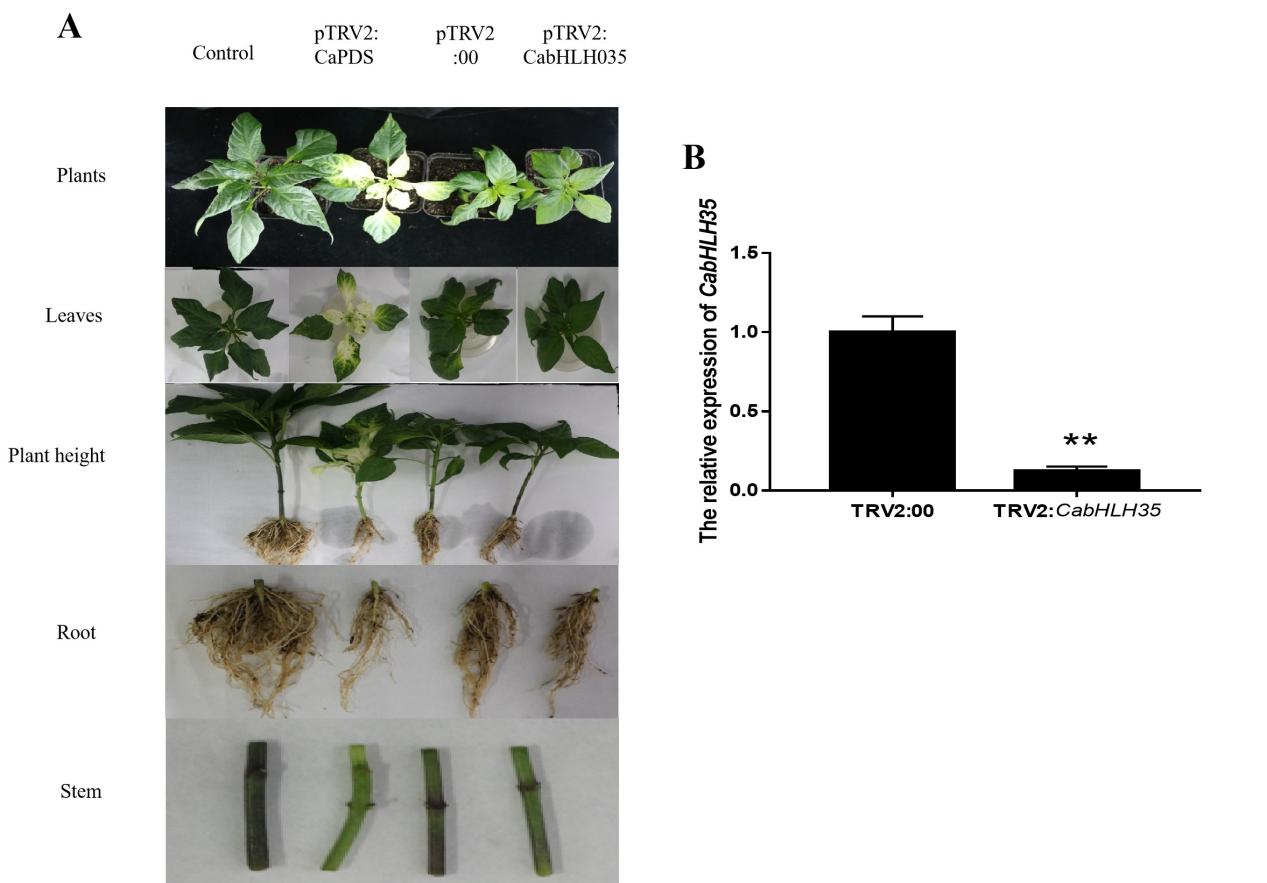


**Figure S1** Phenotypes and silencing efficiency of *CabHLH035* in silenced and control plants. A, The phenotypes of *CabHLH035*-silenced plants. B, qRT-PCR was used to analyze the *CabHLH03*5 expression of TRV2-*CabHLH035* plants, using. Actin2 was used as an internal control.


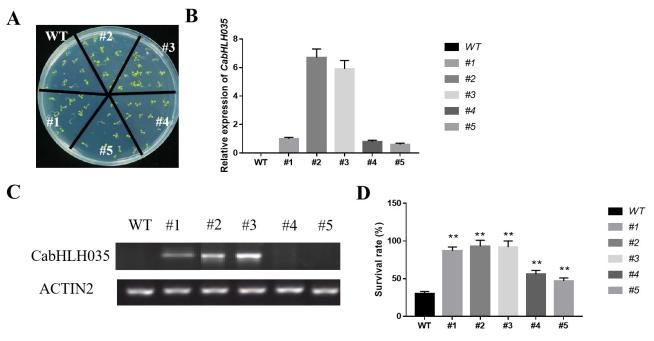
Figure S2

**Figure S2** Characterization of 35S:CabHLH035-GFP of T3 arabidopsis. A, Survival rare of transgenic and WT containing 100mM NaCl; B,C, Expression analysis of CabHLH035-overexpressing transgenic Arabidopsis plants by qRT-PCR; D, survival rates of transgenic and WT under 100mM NaCl.

Table S1. Primers were used for the qRT-PCR.

| Order number | Primer Abbreviation | Primer Sequence (5’- 3’) |
| --- | --- | --- |
| 1 | AtDHAR1-F | GTATCCTGATCCACCTCTCAAG |
| 2 | AtDHAR1 -R | GCTTTGGTGCTAAGCTAAGATC |
| 3 | AtDHAR2-F | AGGCTTTGGTTGATGAGTTAGA |
| 4 | AtDHAR2-R | TGGTAAAGCTTTGGTGCTAAAC |
| 5 | AtDHAR3-F | CTGCGTTAAAGCTTCTATCACC |
| 6 | AtDHAR3-R | TCTCCTCCATTGTCAGTAACAC |
| 7 | AtSOS1-F | ATTTTGATGCAGTCAGTGGATG |
| 8 | AtSOS1-R | GCAAGCAGATTCTAGTCTTTCG |
| 9 | AtSOS2-F | GCGAACTCAATGGGTTTTAAGT |
| 10 | AtSOS2-R | CTTACGTCTACCATGAAAAGCG |
| 11 | AtSOS3-F | CCGGTCCATGAAAAAGTCAAAT |
| 12 | AtSOS3-R | CTCTTTCAATTCTTCTCGCTCG |
| 13 | AtEDR15-F | TTCGACTTGGTACCCTGATTAC |
| 14 | AtEDR15-R | GGAAGAAGATCAGCTACATCGA |
| 15 | AtNHX1-F | ATCACTGCTTTATTGATTGGGC |
| 16 | AtNHX1-R | GCGCCAAAAGCCATAATAGTTA |
| 17 | AtNHX2-F | GTGACGAATAAACTAGCAGCTG |
| 18 | AtNHX2-R | CCATCGATTCTCTTCAAGCAAG |
| 19 | AtDHAR1-F | GTATCCTGATCCACCTCTCAAG |
| 20 | AtDHAR1 -R | GCTTTGGTGCTAAGCTAAGATC |
| 21 | AtDHAR2-F | AGGCTTTGGTTGATGAGTTAGA |
| 22 | AtDHAR2-R | TGGTAAAGCTTTGGTGCTAAAC |
| 23 | AtDHAR3-F | CTGCGTTAAAGCTTCTATCACC |
| 24 | AtDHAR3-R | TCTCCTCCATTGTCAGTAACAC |
| 25 | AtSOS1-F | ATTTTGATGCAGTCAGTGGATG |
| 26 | AtSOS1-R | GCAAGCAGATTCTAGTCTTTCG |
| 27 | AtActin1-F | GTCTGGATTGGAGGGTC |
| 28 | AtActin1-R | TGAGAAATGGTCGGAAA |
| 29 | AtActin2-F | GGTAACATTGTGCTCAGTGGTGG |
| 30 | AtActin2-R | AACGACCTTAATCTTCATGCTGC |
| 31 | CaSOS1-F | GTTCGTGTCTCGTTTCCGC |
| 32 | CaSOS1-R | TCAAATCGGTCTGAACAGCATC |
| 33 | CaHKT2-1-F | GGCATTCATATCAGTTCAGTTTGT |
| 34 | CaHKT2-1-R | TTATCAACAGGCAAAAAAGTAGTAGAG |
| 35 | CaACO-F | ACTTCTTCGCCGACGCCAAG |
| 36 | CaACO-R | GCCAACACATTCACCAGAGCATC |
| 37 | CaNPR1-F | GCCGTGAAGATGTGGG |
| 38 | CaNPR1-R | TGAGTTACGCCAGA |
| 39 | CaABR1-F | ATGACAGGCACAACAGAAGAAAAT |
| 40 | CaABR1-R | AATAAGTTATGACAAGAGCCATTTT |
| 41 | CaDEF1-F | GTGAGGAAGAAGTTTGAAAGAAAGTAC |
| 42 | CaDEF1-R | TGCACAGCACTATCATTGCATACAATTC |
| 43 | CaSOS2-F | AATGACATTGAGGATGCAT |
| 44 | CaSOS2-R | GAAACAAATCGAGTTTG |
| 45 | CaSOS3-F | CAGCTGGCAATTTTCAAGAAT |
| 46 | CaSOS3-R | CTCTCTCAATATAACCA |
| 47 | CaActin2-F | TCCACCTCTTCACTCTCTGCTC |
| 48 | CaActin2-R | TGACCCATCCCTACCATAACAC |
| 49 | CaUBI3-F | TGTCCATCTGCTCTCTGTTG |
| 50 | CaUBI3-R | CACCCCAAGCACAATAAGAC |
| 51 | qPCR-CabHLH035-F | ACAGCCATGTTCTTACCATCGG |
| 52 | qPCR-CabHLH035-R | TCCGGCGAGCTTGAGTCATA |
| 53 | CabHLH035-prokaryotic-CabHLH035 Bam H1 F | CGGGATCC ATGGAAAACATCAGTGAAGACTAC |
| 54 | CabHLH035-prokaryotic-CabHLH035 Sal1 R | GCGTCGAC AGAGCTCATAGGGCTATGTGGATCA |
| 55 | P2 CaSOS1-EMSA-F | CAACACGTCACAACACGTCACAACACGTCACAACACGTCACAACACGTCACAACACGTCA |
| 56 | CaSOS1-EMSA-R | TCTTCTACCATCTTCTACCATCTTCTACCATCTTCTACCATCTTCTACCATCTTCTACCA |
| 57 | CaP5CS-EMSA-F | TAACCATTTGTAACCATTTGTAACCATTTGTAACCATTTGTAACCATTTGTAACCATTTG |
| 58 | CaP5CS-EMSA-R | AGTCTTGGAGAGTCTTGGAGAGTCTTGGAGAGTCTTGGAGAGTCTTGGAGAGTCTTGGAG |
| 59 | Chip-q PCR-CaSOS1-P1 F | CTCCAAGACTATTATAAAAATTTAA |
| 60 | Chip-q PCR-CaSOS1-P1 R | CTCTCTCTATATATATAAGCAAAAT |
| 61 | Chip-q PCR-CaSOS1-P2 F | TATATACTGTATAATAAAATCA |
| 62 | Chip-q PCR-CaSOS1-P2 R | TTTTAAATTTTATCTTGAAACTA |
| 63 | Chip-q PCR-CaSOS1-P3 F | AGGTTAAAAGTAGGCACCTCTA |
| 64 | Chip-q PCR-CaSOS1-P3 R | TAAATATTTTTTTTAATTGAGGT |
| 65 | Chip-q PCR-CaSOS1-P4 F | AAATCACATAAATTAAGATGTG |
| 66 | Chip-q PCR-CaSOS1-P4 R | TTTGTTGTGTTAGATTTTTTTAA |
| 67 | Chip-q PCR-CaP5CS F | GTAGAGTGTTCGAGAAAACAAA |
| 68 | Chip-q PCR-CaP5CS R | ACACAACATGCTAATTATTTACATA |
